# Mortality in Behçet’s Syndrome in a non-endemic population: a 25-year cohort study

**DOI:** 10.1007/s12026-026-09832-9

**Published:** 2026-08-26

**Authors:** Mônica Raquel de Souza Aquino, Barbara Bayeh, Carolina Ejnisman, Fabio Augusto da Rocha Specian, Rafael Bassara Macedo, Pedro Cargnelutti de Araujo, Guilherme Diogo Silva, Gisela Tinone, Rafael Alves Cordeiro, Thiago Quadrante Freitas, Henrique Ayres Mayrink Giardini

**Affiliations:** 1 Division of Rheumatology, Hospital das Clinicas HCFMUSP, Faculdade de Medicina, Universidade de Sao Paulo, Sao Paulo, SP Brazil; 2 Division of Neurology, Faculdade de Medicina, Hospital das Clinicas HCFMUSP, Universidade de Sao Paulo, SP Sao Paulo, Brazil; 3 Department of Neurology, Faculdade de Medicina, Universidade de Sao Paulo, SP Sao Paulo, Brazil

**Keywords:** Behçet's syndrome, Mortality, Survival, Prognosis, Vascular involvement

## Abstract

Mortality in Behçet’s syndrome (BS) has been consistently associated with neurological and vascular involvement in studies from endemic regions. However, data from non-endemic countries remain limited. This study aimed to evaluate long-term mortality and identify factors associated with mortality in a large Brazilian cohort of patients with BS. We conducted a retrospective cohort study including 303 adult patients diagnosed with BS and followed at a tertiary referral center in São Paulo, Brazil, between January 2000 and December 2024. Demographic, clinical, comorbidity, and treatment data were collected. Mortality rates were assessed, and factors associated with mortality were analyzed using unadjusted Cox proportional hazards models, with hazard ratios (HRs) and 95% confidence intervals (CIs). Among the 303 patients included, 61.6% were female, with a median follow-up of 117 months (IQR 55–184). A total of 162 patients (53.4%) were lost to follow-up. Eight deaths (2.6%) occurred during the study period. Given the limited number of events, only exploratory, unadjusted analyses were performed. Pulmonary artery aneurysm (HR 28.44, 95% CI 3.32–243.52; p = 0.002), central nervous system (CNS) parenchymal involvement (HR 6.81, 95% CI 1.65–28.06; p = 0.008), diabetes mellitus (HR 6.72, 95% CI 1.67–27.03; p = 0.007), and malignancy (HR 8.81, 95% CI 2.02–38.50; p = 0.004) were associated with increased mortality. Our exploratory analyses suggest that vascular and neurological involvement, particularly pulmonary artery aneurysm and CNS parenchymal disease, may be associated with increased mortality in our population.

## Introduction

Behçet’s syndrome (BS) is a chronic, relapsing, multisystem vasculitis of unknown etiology, characterized by mucocutaneous, ocular, articular, vascular, gastrointestinal, and central nervous system manifestations, and associated with substantial morbidity and mortality [1–3]. The disease affects both sexes, typically begins during the third decade of life, and generally follows a more severe course in men. BS is most prevalent in countries located along the ancient Silk Road, with the highest incidence reported in Turkey [1–3].

Although ocular disease is the most common severe manifestation and a major cause of morbidity, neurological and vascular involvement—particularly rhombencephalitis and pulmonary artery aneurysms—are the manifestations most strongly associated with mortality [4–7]. Mortality is especially increased among young men during the early stages of the disease and tends to decline over time [5–7]. In addition, a higher frequency of disease flares has been associated with poorer survival outcomes [7].

Beyond disease-related complications, patients with BS are also at increased risk of death from infections, often related to immunosuppressive treatment, as well as from malignancies and cardiovascular diseases [6–9]. Furthermore, a recent meta-analysis demonstrated an association between BS and metabolic comorbidities, including type 2 diabetes mellitus, hypertension, and dyslipidemia, all of which are well-established contributors to cardiovascular mortality in the general population [10].

BS is considered a rare disease in South America, a region regarded as non-endemic for the condition [11]. Nevertheless, a recent study evaluating the epidemiology of systemic vasculitides in tertiary referral centers in Southeastern Brazil identified BS as the most frequently diagnosed vasculitis [12]. Despite the growing recognition of BS in South America, longitudinal data on mortality and its determinants remain scarce. This knowledge gap is particularly relevant because genetic background, healthcare access, and treatment strategies may differ substantially from those observed in classical endemic regions.

Therefore, this study aimed to evaluate long-term mortality and identify factors associated with death in a large Brazilian cohort of patients with Behçet’s syndrome.

## Materials and methods

We conducted a retrospective cohort study of patients with BS aged ≥ 18 years who fulfilled the International Criteria for Behçet’s Disease (ICBD) [13] and/or the International Study Group criteria [14], and who were followed for 25 years, between January 2000 and December 2024, at the Rheumatology Division of the Hospital das Clínicas, University of São Paulo (HC-FMUSP), Brazil. Exclusion criteria included the presence of other systemic inflammatory comorbidities, such as inflammatory bowel disease, connective tissue diseases, or other vasculitis.

The study was approved by the local Ethics Committee (Comissão de Ética para Análise de Projetos de Pesquisa – CAPPesq; CAAE: 59466622.8.0000.0068). All procedures were conducted in accordance with the ethical standards of the institutional research committee and with the Declaration of Helsinki.

Demographic, clinical, and treatment-related data were collected from medical records. Demographic variables included age at symptom onset, age at diagnosis and sex.

Clinical manifestations were categorized as mucocutaneous (oral ulcers, genital ulcers, acne, pseudofolliculitis, erythema nodosum, and skin ulcers), articular (arthritis), vascular (deep vein thrombosis, pulmonary embolism, thrombophlebitis, vena cava thrombosis, suprahepatic thrombosis, portal/splenic/mesenteric thrombosis, central venous thrombosis, pulmonary artery aneurysm, peripheral arterial aneurysms, aortic aneurysm, coronary aneurysm, and arterial occlusion), ophthalmologic (anterior uveitis, intermediate uveitis, posterior uveitis/retinal vasculitis, panuveitis, and non-specified uveitis), neurological (central nervous system parenchymal involvement and aseptic meningitis), and gastrointestinal involvement (ulcers, aphthous ulcers, erosions, perforation, or obstruction occurring anywhere along the gastrointestinal tract, from the esophagus to the anus). Composite variables, including venous thrombotic events, arterial events, any uveitis, and neurological manifestations, were also analyzed.

The mucocutaneous and articular manifestations were assessed during follow-up by the medical team; ocular manifestations were evaluated by ophthalmologic assessment, and neurological and vascular manifestations were confirmed by imaging studies (ultrasound and/or computed tomography angiography or magnetic resonance angiography for vascular manifestations, and magnetic resonance for neurological manifestations) and cerebrospinal fluid (in cases of suspected aseptic meningitis).

Treatment-related variables included the use, during any time of follow up, of glucocorticoids, colchicine, conventional immunosuppressants (azathioprine, cyclophosphamide, cyclosporine, methotrexate, mycophenolate mofetil, and chlorambucil), and biologic agents (infliximab, adalimumab, golimumab, certolizumab, etanercept, ustekinumab, secukinumab, and tocilizumab). Additional grouped variables included immunosuppressant use, biologic therapy, and alkylating agents.

Comorbidities included smoking, alcohol use, diabetes mellitus, systemic hypertension, dyslipidemia, obesity (BMI ≥ 30 kg/m²), fibromyalgia, and cancer.

The primary outcome was all-cause mortality. We also evaluated the causes of death.

Patients were considered lost to follow-up if they had not attended any outpatient visit during the 12 months preceding the end of the study period (December 2024). Vital status was determined exclusively from our institutional medical records, as linkage with national mortality registries was not available.

Categorical variables were expressed as absolute and relative frequencies, and continuous variables as mean ± standard deviation (SD). Because only eight deaths occurred during follow-up, associations between clinical variables and mortality were evaluated using unadjusted Cox proportional hazards regression models, with results expressed as hazard ratios (HRs) and 95% confidence intervals (CIs). Given the limited number of events, multivariable analyses were not performed because they would have produced unstable estimates. Therefore, these analyses should be considered exploratory and hypothesis-generating, and the possibility of residual confounding should be acknowledged when interpreting the results. HRs were not estimated for variables with zero events in one of the groups. Missing data were handled using complete-case analysis.

All analyses were conducted using IBM SPSS Statistics version 22.0, with a two-sided significance level of 0.05.

## Results

A total of 369 patients were initially evaluated, of whom 303 were included. 61.6% were female (*n* = 186) (Table 1). The median follow-up time was 117 months (interquartile range [IQR] 55–184). The causes of death were disease activity (*n* = 4), malignancy (*n* = 3), and undetermined causes (*n* = 1).

**Table 1:** Baseline demographic characteristics according to mortality status

Variable	Category	No death (*n* = 295)	Death (*n* = 8)	HR (95% CI)	*p*-value
Age at symptom onset (years)	Mean ± SD	27.3 ± 11.5	28.5 ± 9.8	1.01 (0.95–1.08)	0.791
Age at diagnosis (years)	Mean ± SD	31.5 ± 10.4	33.4 ± 8.0	1.02 (0.95–1.09)	0.662
Sex	Male, n (%)	114 (97.4)	3 (2.6)	1.00 (ref)	
	Female, n (%)	181 (97.3)	5 (2.7)	1.08 (0.26–4.53)	0.918

Baseline demographic characteristics of patients with Behçet’s syndrome stratified by mortality status during follow-up. Continuous variables are presented as mean ± standard deviation (SD), and categorical variables as absolute and relative frequencies. Associations with mortality were evaluated using unadjusted Cox proportional hazards models

Abbreviations: *HR* = hazard ratio, *CI* = confidence interval, *SD* = standard deviation

The mean age at diagnosis was 31.5 ± 10.4 years among survivors and 33.4 ± 8.0 years among non-survivors (*p* = 0.662) (Table 1). The mean age at death was 47.6 ± 14.1 years. The mean interval between BS diagnosis and death was 14.6 ± 10.3 years. A total of 162 patients (53.4%) were lost to follow-up.

Given the limited number of events, only exploratory, unadjusted analyses were performed to assess factors associated with mortality. Pulmonary artery aneurysm (HR 28.44, 95% CI 3.32–243.52; *p* = 0.002), central nervous system (CNS) parenchymal involvement (HR 6.81, 95% CI 1.65–28.06; *p* = 0.008), and any neurological manifestations (HR 4.36, 95% CI 1.06–17.08; *p* = 0.041) were associated with mortality (Table 2).

**Table 2:** Clinical manifestations and their association with mortality in patients with Behçet’s syndrome

Variable	No death (*n* = 295), n (%)	Death (*n* = 8), *n* (%)	HR (95% CI)	*p*-value
Oral ulcers	293 (99.3%)	8 (100.0%)	Not estimable	0.896
Genital ulcers	240 (81.4%)	7 (87.5%)	1.47 (0.18–11.94)	0.720
Acne	33 (11.2%)	1 (12.5%)	1.74 (0.21–14.61)	0.609
Pseudofolliculitis	153 (51.9%)	3 (37.5%)	0.42 (0.10–1.76)	0.233
Erythema nodosum	106 (35.9%)	0 (0.0%)	Not estimable	0.177
Skin ulcer	13 (4.4%)	0 (0.0%)	Not estimable	0.721
Arthritis	100 (33.9%)	2 (25.0%)	0.55 (0.11–2.77)	0.466
Vena cava thrombosis	13 (4.4%)	0 (0.0%)	Not estimable	0.692
Suprahepatic thrombosis	3 (1.0%)	0 (0.0%)	Not estimable	0.895
Portal/splenic/mesenteric thrombosis	2 (0.7%)	0 (0.0%)	Not estimable	0.916
Central venous thrombosis	20 (6.8%)	1 (12.5%)	2.24 (0.27–18.60)	0.456
Pulmonary artery aneurysm	1 (0.3%)	1 (12.5%)	28.44 (3.32–243.52)	0.002
Peripheral arterial aneurysms	14 (4.7%)	0 (0.0%)	Not estimable	0.728
Aortic aneurysm	2 (0.7%)	0 (0.0%)	Not estimable	0.858
Coronary aneurysm	1 (0.3%)	0 (0.0%)	Not estimable	0.938
Arterial occlusion	20 (6.8%)	1 (12.5%)	2.53 (0.30–21.15)	0.390
Anterior uveitis	37 (12.5%)	1 (12.5%)	0.73 (0.09–6.11)	0.772
Intermediate uveitis	4 (1.4%)	0 (0.0%)	Not estimable	0.815
Posterior uveitis / retinal vasculitis	56 (19.0%)	1 (12.5%)	0.57 (0.07–4.88)	0.601
Panuveitis	16 (5.4%)	1 (12.5%)	2.09 (0.26–17.08)	0.491
Non-specified uveitis	30 (10.2%)	1 (12.5%)	1.45 (0.18–11.85)	0.729
CNS parenchymal involvement	47 (15.9%)	4 (50.0%)	6.81 (1.65–28.06)	0.008
Aseptic meningitis	22 (7.5%)	0 (0.0%)	Not estimable	0.571
Gastrointestinal involvement	17 (5.8%)	0 (0.0%)	Not estimable	0.590
Venous thrombotic events	81 (27.5%)	4 (50.0%)	2.46 (0.61–9.84)	0.205
Arterial events	28 (9.5%)	2 (25.0%)	4.15 (0.80–21.51)	0.090
Any uveitis	120 (40.7%)	4 (50.0%)	1.25 (0.31–5.04)	0.753
Any neurological manifestation	59 (20.0%)	4 (50.0%)	4.26 (1.06–17.08)	0.041

Frequency of mucocutaneous, articular, vascular, ophthalmologic, neurological, and gastrointestinal manifestations in patients with Behçet’s syndrome according to mortality status. Associations with mortality were assessed using unadjusted Cox proportional hazards models

Abbreviations: *HR* = hazard ratio, *CI* = confidence interval. Not estimable = hazard ratio could not be calculated due to zero events in one of the groups

Regarding treatment exposure, colchicine use was associated with lower mortality (HR 0.08, 95% CI 0.02–0.36; *p* = 0.001). In contrast, cyclophosphamide (HR 20.40, 95% CI 2.38–175.00; *p* = 0.006), infliximab (HR 4.97, 95% CI 1.16–20.44; *p* = 0.031), and the use of any alkylating agents (HR 10.74, 95% CI 1.32–87.34; *p* = 0.026) were associated with increased mortality (Table 3).

**Table 3:** Treatments, comorbidities, and their association with mortality in patients with Behçet’s syndrome

Variable	No death (*n* = 295), *n* (%)	Death (*n* = 8), *n* (%)	HR (95% CI)	*p*-value
Glucocorticoid	254 (86.1%)	8 (100.0%)	Not estimable	0.404
Colchicine	262 (88.8%)	4 (50.0%)	0.08 (0.02–0.36)	0.001
Azathioprine	202 (68.5%)	5 (62.5%)	0.80 (0.19–3.39)	0.764
Cyclophosphamide	93 (31.5%)	7 (87.5%)	20.40 (2.38–175.00)	0.006
Cyclosporine	57 (19.3%)	3 (37.5%)	2.00 (0.48–8.41)	0.344
Methotrexate	53 (18.0%)	0 (0.0%)	0.04 (0.00–68.45)	0.387
Mycophenolate mofetil	47 (15.9%)	3 (37.5%)	3.25 (0.75–13.87)	0.115
Infliximab	36 (12.2%)	3 (37.5%)	4.87 (1.16–20.44)	0.031
Adalimumab	21 (7.1%)	0 (0.0%)	Not estimable	0.581
Golimumab	3 (1.0%)	0 (0.0%)	Not estimable	0.828
Certolizumab	3 (1.0%)	0 (0.0%)	Not estimable	0.806
Etanercept	3 (1.0%)	0 (0.0%)	Not estimable	0.852
Tocilizumab	5 (1.7%)	0 (0.0%)	Not estimable	0.811
Chlorambucil	32 (10.8%)	1 (12.5%)	0.73 (0.09–6.14)	0.773
Any immunosuppressants	211 (71.5%)	7 (87.5%)	2.63 (0.32–21.54)	0.367
Any biologic therapy	48 (16.3%)	3 (37.5%)	3.31 (0.79–13.87)	0.102
Any alkylating agent	111 (37.6%)	7 (87.5%)	10.74 (1.32–87.34)	0.026
Diabetes mellitus	35 (11.9%)	4 (50.0%)	6.72 (1.67–27.03)	0.007
Systemic hypertension	91 (30.8%)	4 (50.0%)	1.82 (0.46–7.32)	0.396
Dyslipidemia	94 (31.9%)	5 (62.5%)	2.31 (0.54–9.96)	0.260
Obesity (BMI ≥ 30)	47 (15.9%)	3 (37.5%)	2.49 (0.59–10.52)	0.214
Fibromyalgia	35 (11.9%)	1 (12.5%)	0.81 (0.10–6.67)	0.846
Cancer	11 (3.7%)	3 (37.5%)	8.81 (2.02–38.50)	0.004
Smoking	48 (16.3%)	1 (12.5%)	0.79 (0.10–6.41)	0.823
Alcohol use	14 (4.7%)	1 (12.5%)	4.44 (0.53–37.13)	0.169

Use of glucocorticoid, colchicine, immunosuppressive and biologic therapies, as well as the prevalence of comorbidities, in patients with Behçet’s syndrome according to mortality status. Associations with mortality were evaluated using unadjusted Cox proportional hazards regression models. Abbreviations: *HR* = hazard ratio, *CI* = confidence interval, *BMI* = body mass index. Not estimable = hazard ratio could not be calculated due to zero events in one of the groups

Among comorbidities, diabetes mellitus (HR 6.72, 95% CI 1.67–27.03; *p* = 0.007) and cancer (HR 8.81, 95% CI 2.02–38.50; *p* = 0.004) were associated with increased mortality, whereas smoking, alcohol use, hypertension, dyslipidemia, obesity, and fibromyalgia, were not (Table 3).

No significant associations were observed for demographic variables.

## Discussion

To our knowledge, this is the largest cohort and the study with the longest follow-up of patients with BS conducted in South America, a non-endemic region for the disease. However, our study has several important limitations. The high rate of loss to follow-up and, in particular, the very small number of deaths, restricted the analyses to exploratory, unadjusted Cox models. Consequently, several hazard ratio estimates were imprecise, as reflected by their wide confidence intervals, and should be interpreted with caution. Moreover, because multivariable analyses could not be performed, residual confounding cannot be excluded, and the observed associations should not be interpreted as independent predictors of mortality. Accordingly, our findings should be considered hypothesis-generating and suggest that vascular and neurological involvement may be among the clinical features associated with increased mortality in our population.

The exploratory association observed between pulmonary artery aneurysm and central nervous system involvement and increased mortality in our cohort is consistent with previous studies suggesting that vascular and neurological involvement are the disease manifestations most strongly associated with mortality in patients with BS [5–9, 15, 16]. Similarly, the study by Kural-Seyahi et al. [5], one of the largest and longest cohorts conducted in Turkey, a highly endemic region for the disease, identified pulmonary artery aneurysm and neurologic involvement as the main factors associated with mortality. However, unlike Kural-Seyahi et al. [5], we did not observe an association between mortality and male sex or younger age.”

The exploratory associations observed between treatment exposure and mortality should be interpreted with particular caution because they are highly susceptible to confounding by indication. Cyclophosphamide, infliximab, and other alkylating agents are typically reserved for patients with severe BS, particularly those with vascular and neurological involvement, which are themselves the manifestations most strongly associated with mortality [17]. Therefore, these treatment associations are more likely to reflect underlying disease severity than any direct effect of the therapies themselves. Conversely, colchicine is generally prescribed for patients with milder disease phenotypes [17], and its apparent protective association most likely reflects the lower baseline risk of these patients rather than a true protective effect. Although colchicine has been associated with a reduction in cardiovascular events in patients with ischemic heart disease [18, 19], a specific protective effect on mortality in BS has not been established.

Regarding comorbidities, the exploratory associations observed between diabetes, malignancy, and increased mortality in our cohort are biologically plausible, as both conditions are major causes of death worldwide [20]. A recent meta-analysis demonstrated an increased prevalence of metabolic syndrome, including diabetes, among patients with BS [10]. The authors hypothesized that inflammatory cytokines, such as TNF and interleukin-6, which are elevated in BS, may contribute to insulin resistance, thereby increasing metabolic risk in this population. Similarly, previous studies [6, 7] have identified malignancy as one of the leading causes of death among patients with BS, and a meta-analysis [21] demonstrated an increased incidence of malignancy, particularly hematological cancers, although substantial heterogeneity was observed across the included studies.

Our study has several strengths, including a well-characterized cohort, long-term follow-up, and a comprehensive assessment of clinical manifestations and treatment exposure. However, our findings should be interpreted with caution. First, the very small number of deaths limited the analyses to exploratory, unadjusted models, resulting in imprecise estimates with wide confidence intervals. Second, more than half of the cohort was lost to follow-up, raising the possibility that mortality was underestimated. Because vital status was determined exclusively from institutional medical records and no linkage with a national mortality registry was available, deaths occurring outside our institution may not have been captured. Several factors may have contributed to the high rate of loss to follow-up. Brazil is a large country, and some patients may have relocated or experienced difficulties accessing our tertiary referral center. Patients with milder disease may have transitioned their care to local healthcare providers, whereas some patients with more severe disease may have died or been treated elsewhere. In addition, the COVID-19 pandemic may have contributed to interruptions in follow-up. However, these explanations remain speculative because the reasons for loss to follow-up were not systematically recorded. Future prospective studies incorporating linkage with national mortality databases are warranted to better ascertain vital status, minimize outcome misclassification, and more accurately define predictors of mortality in BS.

Understanding factors potentially associated with mortality in BS in a non-endemic country, such as Brazil, may have important clinical implications. Recently, a randomized clinical trial [22] demonstrated the superiority of infliximab over cyclophosphamide in patients with vascular and neurological BS, and infliximab is now considered first-line therapy for these manifestations [23]. However, infliximab is not approved for BS, and access to this therapy remains limited in our country. Our findings should be considered hypothesis-generating and underscore the need for prospective studies to validate the factors potentially associated with mortality. If confirmed, these findings could contribute to improved risk stratification and support earlier recognition and more intensive management of patients with severe vascular and neurological disease.

In conclusion, our exploratory findings suggest that vascular and neurological involvement, particularly pulmonary artery aneurysm and central nervous system disease, may be among the main clinical manifestations associated with increased mortality in our population. However, given the limited number of events and the unadjusted nature of the analyses, these results should be interpreted as hypothesis-generating. Future prospective, multicenter studies with larger sample sizes are needed to better define independent predictors of mortality and optimize risk stratification in BS in non-endemic settings.

## Data Availability

The datasets generated and/or analyzed during the current study are not publicly available due to patient confidentiality restrictions but are available from the corresponding author on reasonable request.
